# A century of innovative spirit and an optical scientist’s pursuit

**DOI:** 10.1038/s41377-026-02310-9

**Published:** 2026-05-26

**Authors:** Ji Wang

**Affiliations:** Westlake University, No. 600 Dunyu Road, Xihu District, 310030 Hangzhou, Zhejiang China

**Keywords:** Optics and photonics, Applied optics

## Abstract

Five score years ago, Mr. Po-ling Chang, founder of Nankai University in China, established its timeless motto: “**Dedication to Public Interests, Acquisition of All-Round Capability, and Aspiration for Progress with Each Passing Day**.” Today, Gui-Geng Liu, an alumnus of the 2017 Physics Po-ling Program (named after the founder of Nankai University, Mr. Po-ling Chang), has made groundbreaking contributions to topological physics, including the realization of the first three-dimensional Chern insulator and the photonic axion insulator. He is currently an independent Principal Investigator (PI) and doctoral supervisor at the School of Engineering, Westlake University. He has published in top-tier international journals, including *Nature*^1^, *Physical Review Letters*^2^, and *Science*^3^. His research was recognized as one of the Top 10 Social Impact Events in China’s Optics in 2022. In this edition of “Light People”, I am pleased to feature Professor Gui-Geng Liu as he shares his personal journey and growth in the field.

**Figure:**
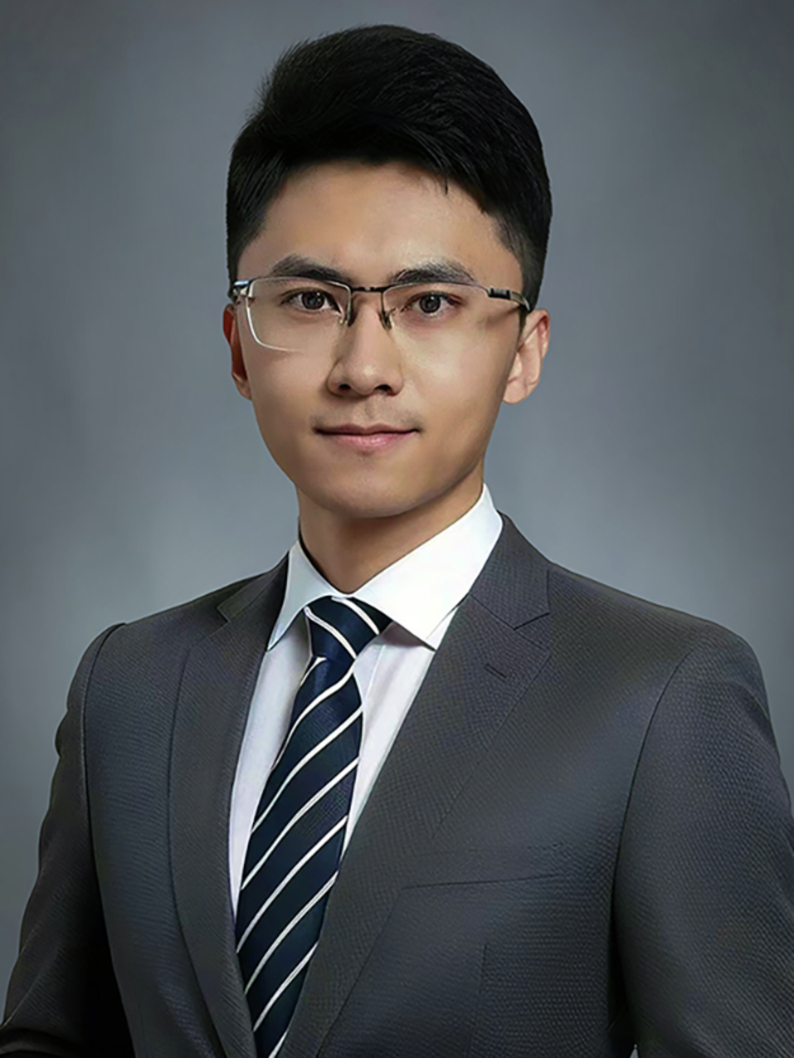

**Short Bio:** Gui-Geng Liu received his B.S. degree from the Po-ling Class, School of Physics, Nankai University in 2017, and his Ph.D. degree from the School of Physical and Mathematical Sciences, Nanyang Technological University (NTU) in 2023. After graduation, he continued his postdoctoral research at NTU. In 2024, he joined Westlake University as a full-time faculty member, serving as an Assistant Professor in the School of Engineering (Independent PI, Ph.D. Supervisor), and was awarded funding under the National High-Level Talent Program for Young Overseas Scholars. His honors and awards include: “Grand Prize of the National College Students’ Innovation and Entrepreneurship Training Program” (2017), “Tianjin Municipal Excellent Graduation Design” (2017), “Chinese Government Award for Outstanding Self-Financed Students Abroad” (2021), “Gold Award for Outstanding Ph.D. Thesis” by the Materials Research Society of Singapore (2023), “NTU Research Excellence Award of the Year” (2023, sole recipient), “Rising Stars of Light (Finalist Award)” (2024), and “Forbes China 30 Under 30” (2024). He was also featured in “2022 Top 10 Social Impact Events in China’s Optics”.

**1. Are there any memorable childhood stories that sparked your early curiosity about science, and how did these experiences subtly shape your path to becoming a scientist?**

To me, scientific exploration is essentially a process of gaining knowledge and understanding the world. I was born in the countryside, and those carefree growing-up days are an exceptionally precious part of my life. The vast fields granted me ample time and space to explore and experiment with my own interests.

As a young boy, I enjoyed creating a wide range of toys and crafts with my own hands. I also grew various plants and carefully observed their growth from sprouting to fruiting. But what fascinated me most was watching ants. I could squat on the ground for an entire afternoon, observing how they divided labor and cooperated to carry food many times their body size back to their nest. Looking back, ants have taught me far more profoundly than any textbook ever could: their perseverance in overcoming difficulties, their knack for breaking down daunting goals and massive food into manageable pieces, and their extraordinary teamwork that turns countless small individuals into an unstoppable collective force. These qualities have subtly become the vital spiritual foundation of my scientific research career.

What truly helped me shape a systematic understanding of science was a book I read in primary school: *A Concise History of Science and Technology*. To this day, I still vividly recall the exhilaration I felt while reading it. With a grand narrative perspective, the book traced the entire journey of humanity’s scientific exploration and comprehension of the world from the dawn of civilization to the present. It was the first time I had truly realized that scientific research is a great relay race across time and space—and that each one of us has the potential to become part of this journey. Since then, the question “What are the fundamental laws governing the world?” has taken root in my mind.

**Figure:**
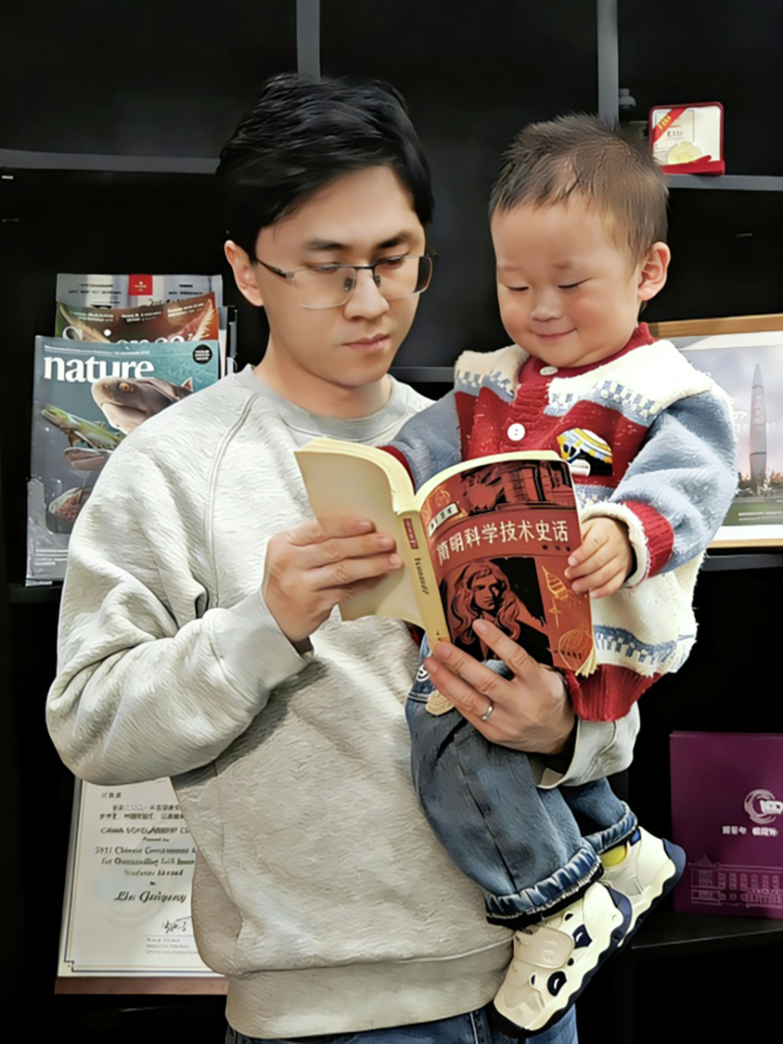
**Dr. Gui-Geng Liu and his son are reading the book** ***A Concise History of Science and Technology***

After I began to study physics in middle school, my desire to explore the scientific world grew stronger. Being able to explain complex natural phenomena with concise and elegant physical principles brought me a kind of intellectual satisfaction and joy that nothing else could replace. Like many young boys who once dreamed of becoming scientists, I am fortunate to uphold this original aspiration to this day, gradually turning my dream into a career and making the exploration of the unknown my lifelong pursuit. Interestingly, despite the passing years, I have found that my curiosity about the world has not changed much from the days when I squatted on the ground watching ants. The only difference is that today I am able to lead a team and make my own modest contribution to the great cause of “exploring the world”.

**2. Your academic journey** **—from leading a team to win the Top Prize of Nankai University in China’s National College Students’ Innovation and Entrepreneurship Training Program, to earning your doctoral degree at Nanyang Technological University, and then being named to the “Forbes China 30 Under 30” list—highlights the profound impact of China’s undergraduate education. What constructive features of China’s undergraduate education do you believe have laid the most critical foundation for your academic growth and career success, and how have these experiences shaped your approach to scientific research?**

My childhood and middle school experiences planted the seeds of science in my heart. Yet the true starting point of my academic research career undoubtedly lies in my undergraduate education at Nankai University. It served as the cornerstone of my academic life and laid a solid, core foundation for my later development.

My decision to major in physics was initially driven purely by personal interest, with almost no regard for practical considerations. Upon entering Nankai University, I learned that the Po-ling Class (named after the founder of Nankai University, Mr. Po-ling Chang) in Physics was recruiting students. The mission of the class is clear: to cultivate future scientists. Driven by my passion for scientific research, I applied and was fortunate to be admitted. The Po-ling Class enabled me to complete a crucial transformation during college: a shift in my identity from a “student” to a “prospective researcher”. Here, we were not confined to an ivory tower, merely memorizing formulas and theorems. Instead, we came to fully understand what it truly means to engage in scientific research—from the real professional contexts and career paths of researchers, to daily research routines, grant applications, and the entire process of academic publishing. All this knowledge allowed me to establish a clear direction for my future early in my university studies.

In terms of research training, the “Po-ling Class” model has benefited me for life. All specialized courses were taught in small classes, where what was encouraged was not “memorizing standard answers,” but questioning, discussion, and critical thinking. More importantly, nearly every course integrated hands-on experiments, small-scale research projects, and academic paper writing. This intensive “theory plus practice” model enabled me to master the basic methods and complete workflow of scientific research at an early stage.

The “Po-ling Class” offered specialized research training programs that required us to participate in genuine cutting-edge scientific research. In my freshman year, I joined a research group led by Professor Chenghou Tu and Professor Huitian Wang at our college. Apart from my coursework, I devoted nearly all my energy to laboratory work, conducting research alongside graduate students. Both professors are exemplary traditional scholars with extremely rigorous academic standards, and their influence on my research habits has been profound. In the lab, I not only acquired knowledge and technical skills; more importantly, I got an early taste of the various challenges that arose in scientific research: the excitement of successful experiments, the frustration of repeated failures, the confusion of hitting research bottlenecks, and even self-doubt about my own capabilities. These experiences had equipped me with resilience and problem-solving abilities long before I started conducting independent research.

I believe that involving undergraduate students in research training is not jumping the gun or an attempt to run before they can walk, but rather a crucial step in applying classroom knowledge immediately to cutting-edge scientific issues, bridging textbook learning and real-world practice. Above all, this process cultivates the ability to solve problems. It also enables students to judge at an early stage whether scientific research is truly right for them and whether they have a real passion for it.

Encouragingly, this model has become increasingly prevalent among universities in China. At Westlake University in particular, every undergraduate student is assigned an academic mentor upon enrollment to support their growth, allowing them to engage directly with cutting-edge research. Currently, several undergraduate students in our lab are far more outstanding than I was at their age and possess a much higher starting point. I sincerely hope that my experiences and insights can help them go further on their academic journey.

**Figure:**
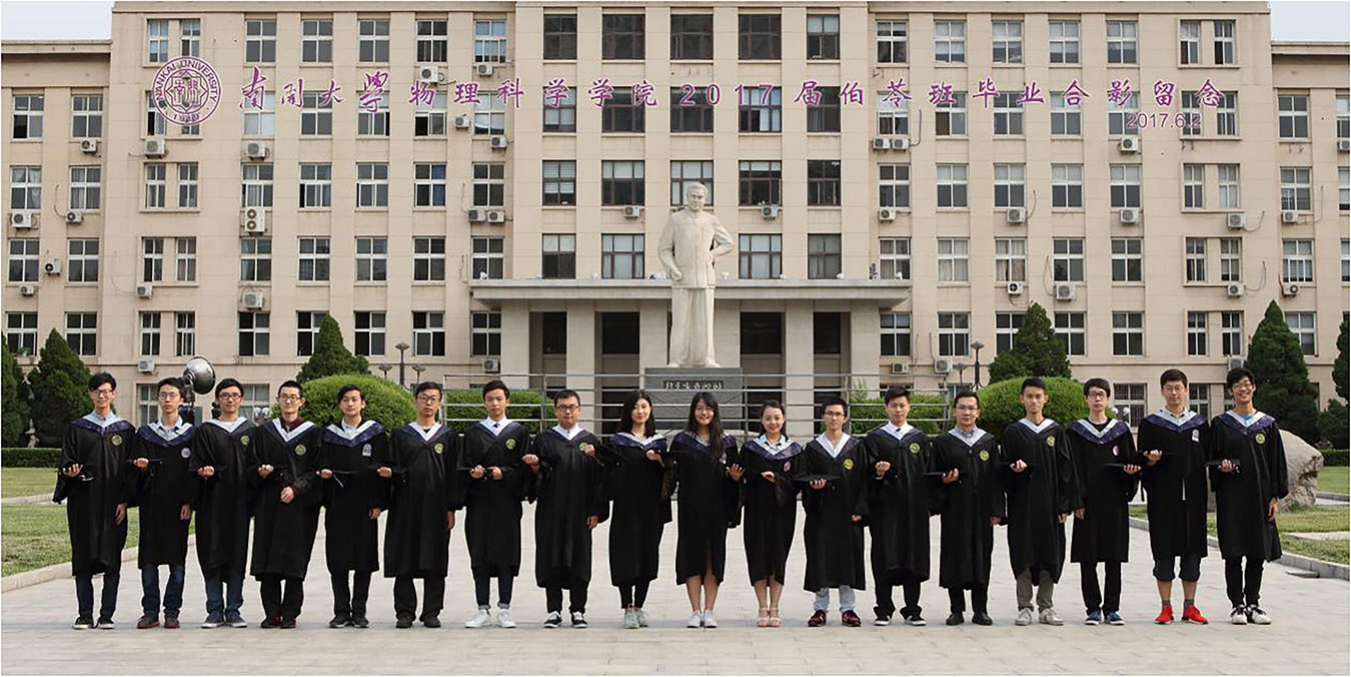
**Gui-Geng Liu (seventh from right) in the 2017 graduation photo of the Po-ling Class, School of Physics, Nankai University**

**3. What initially sparked your interest in the field of topological photonics, and how has this passion evolved throughout your academic career?**

My interest in topological photonics actually grew naturally out of my research experience as an undergraduate. During my undergraduate research, I focused primarily on structured light fields—simply put, shaping light into desired forms by controlling its degrees of freedom, such as phase and polarization. Topological photonics, by contrast, describes a fundamental, global property of light that is robust against local perturbations. This shift from a local perspective to a global one has fascinated me deeply. Moreover, topological photonics carries profound physical significance, enabling the discovery of new topological states of matter, while also holding great promise for applications in the design of novel photonic devices.

After graduating from Nankai University, I joined the research group of Professor Baile Zhang and Professor Yidong Chong at Nanyang Technological University. Both supervisors are leading experts in topological optics, and with my previous research background, I was able to quickly immerse myself in research and independently conduct research projects. At that time, topological photonics was undergoing rapid development. Most studies in the field focused on structures based on non-magnetic materials. Such systems are easy to realize and exhibit rich phenomena, and thus naturally attract the most extensive attention. However, I developed a particular interest in magnetic photonic crystals. They feature the “cleanest” and most fundamental physical mechanism: time-reversal symmetry breaking directly gives rise to non-trivial topological properties, with a clear and profound physical picture underlying them, which is expected to yield delicate and novel physical phenomena. During my Ph.D. studies, I devoted most of my efforts to investigating magnetic photonic crystals. Starting from two-dimensional systems, I gradually expanded my research into more complex three-dimensional systems and made much progress. To this day, this direction remains one of the core research areas in my laboratory. Each deeper investigation uncovers new subtle insights. The journey from the periphery to the mainstream, and from a niche topic to the cutting-edge field, encapsulates the unique charm of scientific exploration.

**Figure:**
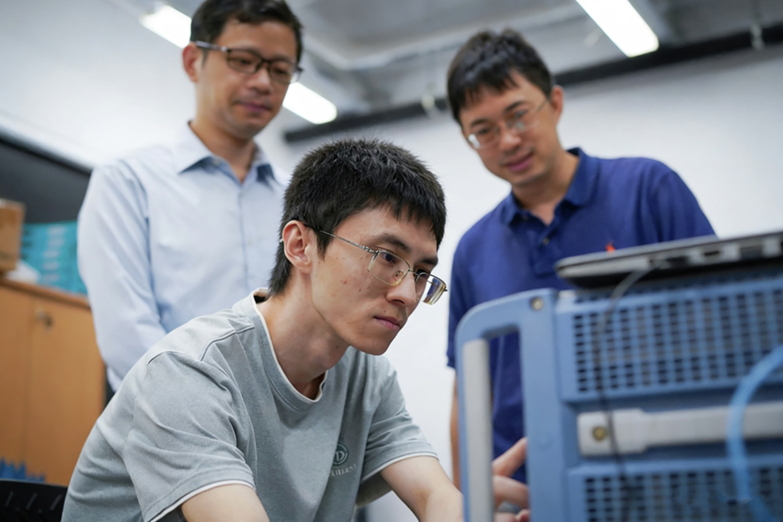
**Dr. Gui-Geng Liu (center), his Ph.D. supervisors Prof. Baile Zhang (back left), and Prof. Yidong Chong (back right), are conducting experiments at Nanyang Technological University**

**4. Looking back at your research on the first three-dimensional Chern insulator, what was the biggest challenge you encountered, and how did you overcome it?**

The greatest challenge in this research does not stem from the abstruseness of theoretical derivations, nor from the precision of experimental designs, nor even from repeated failures during experiments. Rather, it lies in how to uncover truly groundbreaking physical insights hidden within a seemingly mundane, even slightly “uninteresting” research idea.

The original research idea was remarkably simple: stack the prototypical two-dimensional photonic Chern insulator layer by layer to form a three-dimensional system, and observe whether any novel phenomena would emerge. The initial findings were indeed unremarkable: the one-dimensional chiral edge states of the two-dimensional Chern insulator evolved into two-dimensional surface states in the three-dimensional system. This result offered no surprises whatsoever. Later, through ingenious experimental design, I discovered that this three-dimensional photonic crystal could undergo a phase transition from a three-dimensional Chern insulator to a Weyl semimetal, and developed a comprehensive theoretical model to explain this process. This discovery filled an experimental gap, yet it still failed to address our initial core concern: what is the essential difference between a three-dimensional Chern insulator and its two-dimensional counterpart? During the peer-review process of our paper under consideration by *Nature*, the reviewers raised this very question. We seemed to have returned to square one. By then, the project had been underway for three years, and we remained completely stuck.

**Figure:**
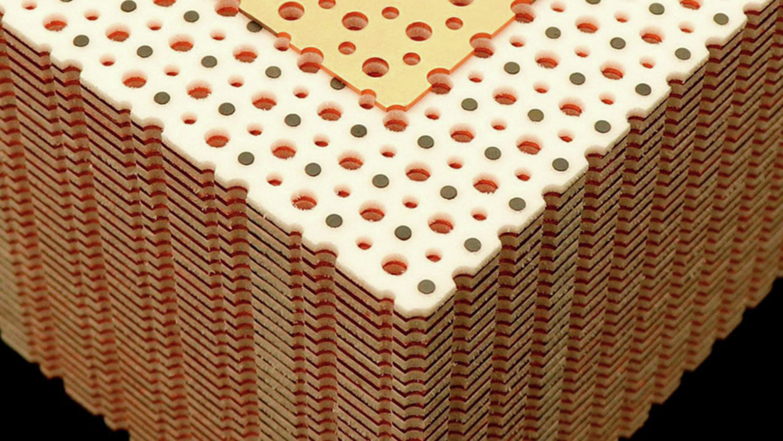
**The three-dimensional Chern insulator sample fabricated by Dr. Gui-Geng Liu**

The real turning point came from a discussion with my supervisor, Professor Baile Zhang. He suggested investigating the interface states between two distinct three-dimensional Chern insulators. I immediately designed structures and carried out simulations, and the results were exciting: topologically protected surface states did exist at the interface. More importantly, I found that these surface states could form intricate and elegant knot and link structures in momentum space. These correspond precisely to the torus knots and torus links investigated in mathematical knot theory, a finding that bridges band topology in materials with structural topology in mathematics. Further investigations revealed that these phenomena arise from the unique “Chern vector” property of three-dimensional Chern insulators—a novel characteristic that distinguishes three-dimensional systems from two-dimensional systems. Both my supervisor and I were delighted by this finding, and eventually the paper was successfully published in *Nature*^1^.

**Figure:**
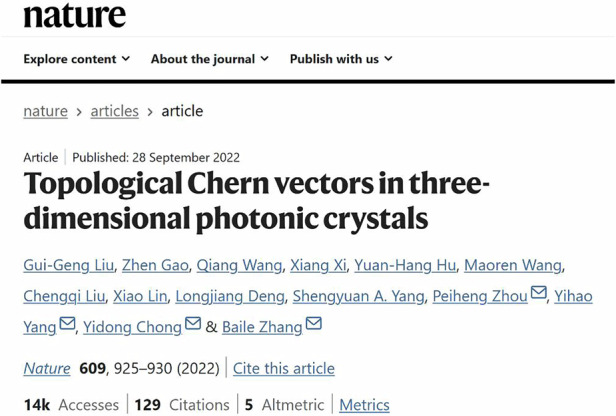
**In 2022, Dr. Gui-Geng Liu published a research paper in the journal** ***Nature***^1^

Looking back on the success of this research, two qualities are crucial: first, resilience and perseverance—most of scientific research is mundane or even bewildering; genuine breakthroughs frequently emerge from these seemingly ordinary days, and only persistence can bring them to light; second, the spirit of openness and collaboration—at the most critical moment, it was the perspectives of my collaborators that inspired new insights. Coincidentally, these are the very qualities I learned from observing ants in my childhood.

**5. In your 2024 paper published in** ***Physical Review Letters***^2^*,* **one of the most prestigious and influential physics journals worldwide, you reported the localization of chiral edge states induced by the non-Hermitian skin effect. Could you briefly outline the key findings, the underlying physical mechanism, and the broader significance of this work to the field of non-Hermitian topological photonics?**

Our study explores a rather counterintuitive phenomenon in non-Hermitian topological systems: chiral edge states, which were long thought to be immune to localization, can actually be controllably localized in space through the non-Hermitian skin effect.

Let me first give a bit of background. The non-Hermitian skin effect arises when gain or loss is introduced into a system, causing a large number of eigenstates to accumulate at the boundary. Chern insulators, in contrast, are among the most fundamental models in topological physics—the quantum anomalous Hall effect being a classic example. In a conventional Hermitian Chern insulator, the bulk-boundary correspondence guarantees the existence of chiral edge states. These states propagate unidirectionally and are protected against backscattering, so they have long been considered robust against spatial localization, regardless of impurities or disorder.

In this work, we brought the non-Hermitian skin effect into a Chern insulator. By carefully designing the spatial profile of loss, we found that chiral edge states can be localized to specific edges or even corners. Concretely, we realized such a non-Hermitian Chern insulator in a magnetic photonic crystal. By incorporating materials with different loss properties, we created point-gap structures in the complex energy spectrum and used point-gap winding numbers to characterize the skin effect. When the winding numbers in both directions are nonzero, the edge states become further confined to the corners of the two-dimensional sample. We proposed a hybrid topological invariant that combines the Chern number (which captures the Hermitian topology) with the point-gap winding numbers (which capture the non-Hermitian skin effect). This invariant provides a unified description of boundary states in both Hermitian and non-Hermitian systems, thereby extending the conventional bulk-boundary correspondence into the non-Hermitian regime.

The significance of this work is twofold. On the fundamental side, our study demonstrates that photonic crystals can serve as a powerful platform for exploring non-Hermitian topology. By realizing a hybrid system that combines the non-Hermitian skin effect with a Chern insulator, we show that the conventional bulk-boundary correspondence—a cornerstone of Hermitian topology—can be extended into the non-Hermitian regime. This highlights the role of photonic systems not only as testbeds for existing theories, but also as arenas for uncovering new topological principles. On the application side, the localized chiral edge states we obtain retain the inherent robustness of Chern insulators while gaining precise spatial confinement. This unique combination makes them well-suited for photonic devices such as topological lasers and robust light harvesting.

**Figure:**
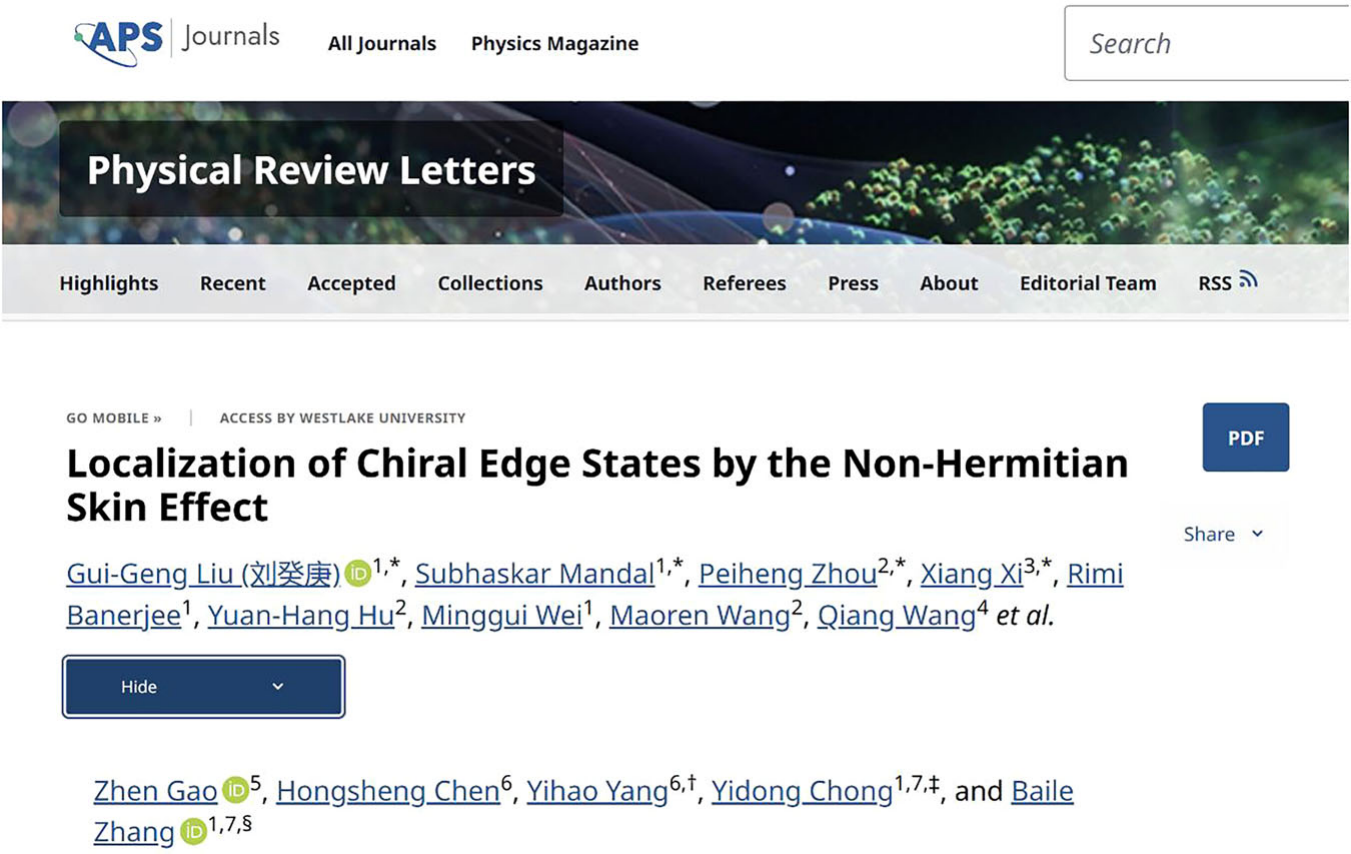
**In 2024, Dr. Gui-Gng Liu published a research paper in the journal** ***Physical Review Letters***^2^

**6. What motivated you to join Westlake University in 2024 rather than other academic institutions, and what do you think makes this university particularly suitable for your research?**

Choosing to join Westlake University in 2024 was a decision I made after careful consideration. The real reason is quite simple: here, I can stay fully devoted to the most genuine pursuit of scientific research, and focus peacefully on the work I truly love and aspire to do.

Westlake University boasts an exceptionally streamlined administrative system, free from cumbersome bureaucratic procedures and empty formalities. This enables us to devote nearly all our time and energy to research. For a young scholar just launching an independent research group, such an environment of “uninterrupted focus on research” is truly invaluable.

Meanwhile, Westlake University brings together a group of leading scholars at the forefront of various disciplines. Whether in basic physics, materials science, or applied engineering, I can quickly find like-minded collaborators. All schools at Westlake University are connected through the Academic Ring, allowing me to quickly access any colleague’s office for in-depth discussions. Many ongoing projects in my lab are being conducted in collaboration with several colleagues. Westlake University is truly an academic community where colleagues on campus can engage in discussions and launch collaborations at any time.

**Figure:**
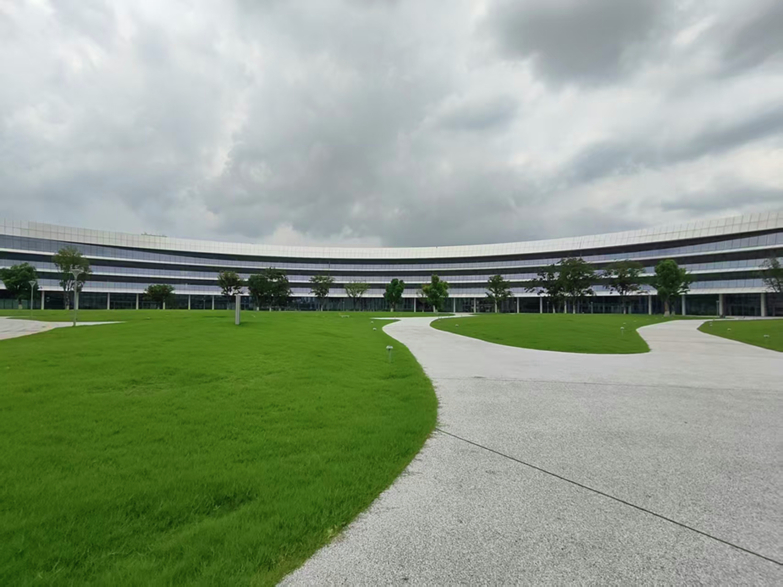
**Westlake University’s Academic Ring**

**7. As a principal investigator (PI) at Westlake University, how do you balance guiding your research team and pursuing your own independent research projects?**

For me, “leading a research team” and “pursuing individual research” are not opposing elements that demand strenuous balancing. Instead, they form an organic whole that integrates and enhances each other. In fact, my research has always been collaborative in nature—and this was true of my past work, just as it remains so today at Westlake University, where my team members are my principal collaborators. Accordingly, no individual research project exists entirely independent of the team.

I share with my team the scientific questions I am pondering, the physical pictures I have tentatively formed, and even fleeting ideas that strike me. I do not treat these ideas as tasks to be assigned; instead, I sincerely invite interested group members to join in. The most crucial principle I always adhere to is that before launching any research project, I ask every member a simple question: “Are you truly passionate about this topic?” Only when students are driven from their hearts to explore will I support them in moving forward. I never push anyone into research they are not interested in. Instead, I help match them with more suitable projects based on their background and strengths.

At the same time, I also encourage students to independently identify research topics. I teach them my full approach to developing research topics: how to read cutting-edge literature efficiently, how to extract meaningful scientific questions from existing research, how to design research plans around those questions, and how to assess a topic’s scientific value and feasibility. In my view, teaching students to think and explore autonomously is far more important than simply helicoptering them. This approach not only helps them truly grow in research but also allows them to experience the joy and sense of accomplishment that come from their own exploration.

This model brings a host of benefits: students grow rapidly, driven by their own interests and gain a strong sense of achievement in the research process; team projects advance efficiently based on shared enthusiasm; and I, together with a group of like-minded partners, turn ideas into reality one by one. More importantly, the new insights and questions raised by students through independent exploration often provide fresh perspectives and inspiration for my own research in return.

**8. Your work on photonic axion insulators has achieved significant breakthroughs. Could you share the most exciting moment during this research process?**

Research on photonic axion insulators bears similarities to that on three-dimensional Chern insulators: both start from seemingly ordinary ideas yet ultimately reveal profound and distinctive underlying physics. Our initial vision was rather simple: to construct an antiferromagnetic photonic crystal and investigate the possible existence of one-dimensional chiral hinge states. This idea can be regarded as a natural extension of my earlier work on three-dimensional Chern insulators. Constrained by the theoretical understanding at that time, we did not deliberately connect this study with the concept of “axion insulators”, even though we intuitively sensed a potential connection.

The real breakthrough stemmed from an in-depth discussion with my collaborators. At that time, my collaborators had designed a purely theoretical photonic crystal model and theoretically calculated its axion field. I shared the preliminary results of our experimental system with them and posed a question: “Could our system also host a quantized axion field?” This inquiry sparked over a year of collaborative research. Together, we derived formulas, performed repeated calculations, and carried out extensive verifications. Eventually, we confirmed that our system indeed possesses a quantized axion field with a half-integer surface Chern number. This indicates that the experimental system we constructed is a genuine axion insulator. At that moment, all the previously scattered experimental findings received a unified and elegant explanation within this theoretical framework. That moment of “sudden enlightenment” is the most cherished experience for researchers. In 2025, this work was successfully published in *Science*^3^. I am truly grateful to my collaborators for their years of relentless effort and unwavering dedication to this research.

**Figure:**
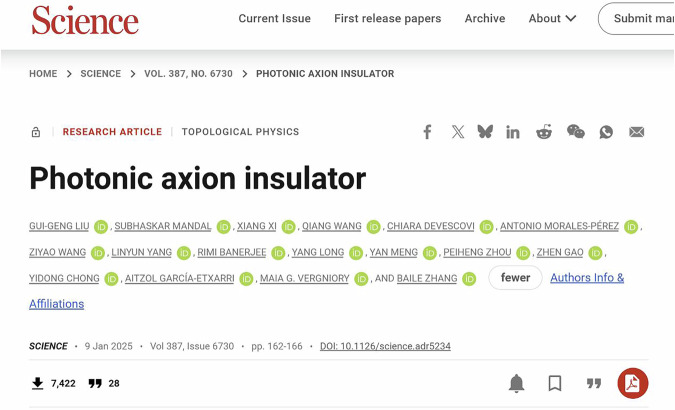
**In 2025, Dr. Gui-Geng Liu published a research paper in the journal** ***Science***^3^

**9. You have been engaged in scientific research for over a decade. How do you sustain innovation and creativity in your research?**

Looking back on my research journey, those seemingly sudden inspirations are simply natural outcomes of long-term accumulation. For me, sustaining innovation mainly lies in three aspects: continuous learning and knowledge building, in-depth thinking, and open discussion.

Learning is the foundation of innovation. My desire for new knowledge and curiosity about the unknown drives me to constantly expand my intellectual boundaries. I not only keep a close eye on cutting-edge developments in topological photonics but also often read research from other disciplines, and many important insights arise from seemingly unrelated fields. When reading key literature, I spend days or even longer studying it thoroughly until I fully grasp its core ideas. While reading, I keep asking: What if we take a different approach? Can this method solve our problems? Most ideas get filtered out; only the most feasible and valuable one becomes the starting point of a new project.

Meanwhile, I devote myself to prolonged, in-depth thinking on fundamental issues. I often ponder difficult questions for years, constantly decomposing, restructuring, and connecting ideas in my mind until a clear physical picture takes shape. The so-called sudden insight is never accidental; it arises when disparate clues suddenly come together after long-term, immersive contemplation.

Besides, open discussion is equally important. As an old saying goes, “Learning alone without exchanges with others will lead to ignorance.” I often exchange ideas with team members and also discuss projects with scholars from diverse fields. Varied perspectives in themselves constitute a source of innovation. Many long-baffling problems often become clear in light-hearted cross-disciplinary discussions.

**10. What are the most important factors you consider when recruiting members for your research team, and what qualities do you value most in young researchers? Looking back on your academic experiences at Nankai University and Nanyang Technological University, how have they shaped the way you mentor students at Westlake University?**

In recruiting team members, my primary consideration is a person’s intrinsic qualities and original aspiration for research—whether they are truly suited for scientific work, and possess the potential to persist in research for the long term and succeed in the long run. The qualities I value most can be summarized as follows, and they are also the golden rules I hold for myself.

First and foremost, maintain strong intrinsic motivation. This drive stems not from external pressure but from an innate desire to explore the unknown and pursue breakthroughs. Highly self-motivated individuals proactively define research objectives, immerse themselves in literature, and conduct experiments. When research encounters bottlenecks, researchers actively seek solutions instead of relying on others. This is the most fundamental quality for researchers and the cornerstone of sustainable progress in scientific research.

Secondly, think independently and have the courage to question. The essence of research lies in exploring the unknown and discovering new laws, not in blindly following authorities or repeating others’ work. I hope team members can think independently, put forward their own opinions on existing theories and findings, dare to question and inquire in research, break free from fixed mindsets, and examine problems from new perspectives. Independent thinking is at the heart of scientific innovation, and the courage to question is the prerequisite for research breakthroughs.

Thirdly, embrace a strong collaborative spirit. Modern scientific research, especially interdisciplinary research, can never be accomplished by a single person working alone; it is the result of close collaboration within a team. I hope team members can collaborate and support one another, actively share their research ideas and findings, and stay open to others’ opinions and suggestions, so as to foster a positive atmosphere where teaching and learning benefit each other and research and thinking reinforce one another.

Above all, stay calm and patient, and never be obsessed with quick success. Major breakthroughs cannot be achieved overnight; they require long-term dedication and perseverance. As the old saying goes, “The last leg of a journey marks the halfway point.” All too often, success is just one tough step away.

The most valuable lessons I took from Nankai University and NTU come down to two things: help young researchers see what science really looks like as early as possible, and then give them the space to focus on what truly matters.

My undergraduate years at Nankai made me realize that the formative years for a researcher are not in graduate school, but during college. In the Po-ling Class, I was not made to wait until graduate school to engage in research. From my first year, I was involved in lab research—not for mere token “research experience,” but actually to work on real research problems, going through real failures and breakthroughs. That experience helped me make a mental shift from “acquiring knowledge” to “doing science” far earlier, and it gave me clarity on my direction before I even set out. That kind of clarity—knowing what you’re getting into before you commit—saves years of wandering, frustration, and second-guessing. My Ph.D. training at NTU showed me something else: the best research environments aren’t about lavish resources, but about cutting away distractions. There, my advisors got straight to the heart of the problems. The lab ran efficiently, without bureaucracy. I poured every ounce of my energy into the science itself and came to understand that great work doesn’t come from piling up resources—it comes from sustained, uninterrupted reflection.

What both schools taught me, in the end, is the same : to train a researcher, first help them understand what research truly demands, and then clear the path so they can give it their full attention. That’s what I hope to create for my students at Westlake—a chance to see science clearly from the start, and an environment where they can pour their energy into the problems that matter most.

**Figure:**
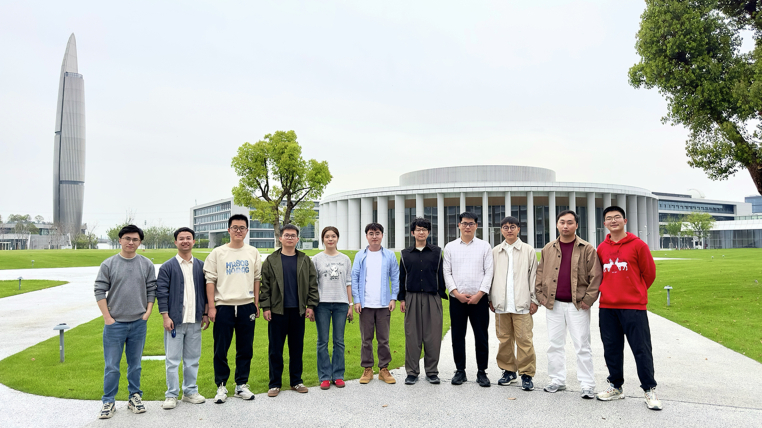
**Prof. Gui-Geng Liu’s research group at Westlake University**

**11. Looking ahead to the next 5-10 years, what are your main research goals, and what impact do you hope your work will have on the academic community and society? Do you plan to publish your findings in** ***Light: Science & Applications*, a top-tier open-access optics journal, jointly published by the Changchun Institute of Optics, Fine Mechanics and Physics, CAS, and** ***Nature*** **Portfolio?**

Over the next 5 to 10 years, my team will focus on photonics, pursuing breakthroughs that extend from the very foundations of physics to functional devices. That vision often brings to my mind the motto of Nankai University, formulated by its founding president, Mr. Po-ling Chang: “Dedication to public interests, acquisition of all-round capability, and aspiration for progress with each passing day.” To me, “dedication to public interests” means keeping society in mind and putting our research to real-world use; “acquisition of all-around capability” speaks to our drive to uncover the fundamental laws of nature while continuously sharpening our ability to explore and innovate; “aspiration for progress with each passing day” captures the spirit of continuous breakthroughs and an unyielding determination to move forward. These ideals align closely with what I strive for in my team’s scientific journey.

At the fundamental level, our work focuses on exploring core physical mechanisms, including topological photonics and non-Hermitian optics. Topological photonics elucidates how light can propagate in a robust, defect-immune manner, while non-Hermitian optics unlocks new regimes of optical control by leveraging gain, loss, and other non-conservative effects. These fields embody profound and largely unexplored physics, with many fundamental questions still awaiting answers. I hope that along these directions, we can not only discover novel phenomena and establish new theoretical frameworks, but also advance our fundamental understanding of light-matter interactions. As for me, fundamental research is invaluable, as it serves as the wellspring of future technological innovation and transformation.

When it comes to applications, my goal is to translate these physical mechanisms into practical photonic chips. I intend to harness the robustness of topological protection and the versatility of non-Hermitian control to develop novel approaches for optical signal manipulation, embedding these fundamental principles directly into on-chip architectures. My ambition extends far beyond laboratory-scale demonstrations: I aim to deploy such chips in real-world scenarios, including optical communications and optical computing. Only by connecting fundamental research to genuine societal needs can we truly honor the ideal of dedicating ourselves to both public service and intellectual advancement.

I am also very much looking forward to publishing our best work in *Light: Science & Applications*. As one of the most internationally influential journals based in China, it offers a wonderful platform for scholarly exchange and stands as a testament to how far Chinese optics has come, from catching up to keeping pace, and even taking the lead in some fields. My team and I will continue to work hard to produce solid, meaningful research and contribute impactful work to this journal.

**12. Beyond academic research, what hobbies or activities do you usually engage in to relax, and how do they help you recharge for your work?**

As for me, scientific research is inherently a pursuit driven by curiosity and passion. Throughout my research journey, what I have experienced most is the joy of exploring the unknown and the fulfillment of realizing my personal value, so I seldom feel the need to relieve pressure intentionally.

In my leisure time beyond research, my greatest comfort is spending quality time with my family, especially playing with my son. Interestingly enough, I have always enjoyed the company of young children. William Wordsworth, the great British Romantic poet, once wrote, “The Child is father of the Man.” I also deeply resonate with the ideal of “returning to the state of infancy” expressed in the Chinese classic *Tao Te Ching* (*The Classic of the Way and Virtue*) —perhaps because my curiosity and sense of wonder about the world have remained largely unchanged since childhood. Watching him explore everything around him with clear, innocent eyes allows me to set aside my busy schedule and find a pure sense of peace and joy.
